# Risk factors of in-stent restenosis in patients with diabetes mellitus after percutaneous coronary intervention: A protocol for systematic review and meta-analysis

**DOI:** 10.1097/MD.0000000000025484

**Published:** 2021-04-16

**Authors:** Suiping Li, Chao Luo, Haimei Chen

**Affiliations:** a Department of Endocrinology; b Department of Neurology; c Department of Hematology, Zhuzhou Central Hospital, Zhuzhou 412000, Hunan Province, China.

**Keywords:** diabetes mellitus, in-stent restenosis, meta-analysis, percutaneous coronary intervention, protocol, risk factors

## Abstract

**Background:**

Percutaneous coronary intervention (PCI) has become one of the effective methods for the treatment of coronary heart disease (CHD). However, it is easy to have in-stent restenosis (ISR), even cardiovascular events after PCI, which affects the therapeutic effects. The incidence of ISR in diabetes mellitus (DM) patients increased by 2 to 4 times. Early identification of the risk factors of ISR in DM patients after PCI may help clinical staff to prevent and intervene as soon as possible, so it is very important to improve the clinical outcomes of DM patients. Although scholars at home and abroad have studied and summarized the risk factors of ISR in DM patients after PCI, the conclusions are different. Therefore, in this study, meta-analysis was used to summarize the risk factors of ISR in DM patients after PCI, and to explore the characteristics of high-risk groups of ISR, thus providing reference for early identification and prevention of ISR.

**Methods:**

We will search related literature from PubMed, Embase, Cochrane Library, Web of Science, China Biology Medicine Database, China National Knowledge Infrastructure, China Science and Technology Journal Database, and Wanfang Database. Eligible studies will be screened based on inclusion criteria. Meanwhile, data extraction, risk of bias assessment, publication bias assessment, subgroup analysis, and quality assessment will be performed. Review Manager Version 5.3 software will be applied for data analysis. Each process is independently conducted by 2 researchers. If there is any objection, it will be submitted to a third researcher for resolution.

**Results:**

We will disseminate the findings of this systematic review and meta-analysis via publications in peer-reviewed journals.

**Conclusions:**

The results of this analysis can be used to generate a risk prediction model and provide an intervention strategy for the occurrence of ISR in DM patients after PCI.

**OSF REGISTRATION NUMBER:**

DOI 10.17605/OSF.IO/WC87Y.

## 1 Introduction

Percutaneous coronary intervention (PCI) has become a major treatment for coronary heart disease (CHD).^[1–3]^ However, a number of large clinical studies have revealed that, from the era of bare metal stents to the widespread clinical application of drug-eluting stents, 3% to 20% of patients still have in-stent restenosis (ISR).^[4]^ At present, some scholars explore ISR from the aspects of drug type, stent type, and implantation technique, and have achieved fruitful results.^[5,6]^ However, with the rapid development of PCI and the wide application of intravascular ultrasound, the indications of PCI are expanding, while the incidence of ISR is still increasing. However, at present, the mechanism of ISR is not clear, and many factors are involved in the occurrence and development of ISR.^[7]^

At present, it is considered that bare metal stent, history of DM, residual stenosis after PCI, diameter, and length of stent are the influencing factors of ISR after PCI.^[8]^ DM patients have more complex coronary artery lesions, usually accompanied by dyslipidemia and abnormalities in the blood coagulation system.^[9,10]^ DM patients are likely to develop ISR due to excessive intimal hyperplasia, excessive hemagglutination, increased inflammation, endothelial dysfunction, and complications.^[11,12]^ Park et al proposed that the history of DM is an independent risk factor of ISR in patients after PCI.^[13]^ Some studies have proved that the risk of developing ISR in patients with diabetes is 2 to 4 times higher than that in ordinary patients.^[14–17]^ The related predictors of ISR after PCI in DM patients are not clear. Therefore, as a high-risk group, DM patients have attracted more and more researchers’ attentions.

At present, there is no unified understanding of the pathogenesis, epidemiological status, diagnosis, and treatment of ISR in DM patients after PCI, and the research results of its risk factors are not same. In order to synthesize the results of previous studies,^[18–23]^ this paper makes a meta-analysis on the risk factors of ISR in DM patients after PCI for the identification the risk factors of ISR in DM patients after PCI, thus providing scientific basis for clinical prevention of ISR in DM patients after PCI.

## 2 Methods

### 2.1 Study registration

This protocol has been registered on Open Science Framework grant number: DOI 10.17605/OSF.IO/WC87Y (https://osf.io/wc87y). This report will be based on the preferred reporting items for systematic review and meta-analysis protocols.^[24]^

### 2.2 Eligibility criteria

Inclusion criteria:

1) Study design: We will include all observational studies (case–control study, cohort study, prospective study, etc) to analyze the correlation between risk factors and ISR in DM patients after PCI;
2) Participants: 18 years and above adults with the ISR in DM patients after PCI will be included;
3) Diagnosis of ISR: ISR, defined as coronary angiography, confirmed that the diameter of the lumen in the stent implantation segment and the proximal and distal 5 mm segments of the stent is more than 50% narrow;
4) Outcomes: The results of the study involve the specific values of odds ratio (OR) and 95%confidence interval (95%CI) of risk factors.

Exclusion criteria:

1) The full text cannot be obtained normally or the extracted data are affected;
2) Repeatedly published literatures;
3) Review, systematic review, conference, animal experiments, and other literatures.

### 2.3 Search strategy

Electronic databases include PubMed, Embase, Cochrane Library, Web of Science, China Biology Medicine Database, China National Knowledge Infrastructure, China Science and Technology Journal Database, and Wanfang Database. The search terms have diabetes mellitus, ISR, in-stent restenosis, PCI, CHD, risk factor, risk assessment, multivariate analysis, and multivariable logistic regression. The search dates are from the establishment of the database to February 2021. These search terms are summarized in Table 1.

**Table 1:** Search strategy in PubMed database.

Number	Search terms
#1	Diabetes Mellitus[MeSH]
#2	Coronary Restenosis[MeSH]
#3	Coronary Restenoses[Title/Abstract]
#4	Restenoses, Coronary[Title/Abstract]
#5	Restenosis, Coronary[Title/Abstract]
#6	In-stent restenosis[Title/Abstract]
#7	or/2–6
#8	Percutaneous Coronary Intervention[MeSH]
#9	Percutaneous Coronary Revascularization[Title/Abstract]
#10	Coronary Intervention, Percutaneous[Title/Abstract]
#11	Coronary Interventions, Percutaneous[Title/Abstract]
#12	Coronary Revascularization, Percutaneous[Title/Abstract]
#13	Coronary Revascularizations, Percutaneous[Title/Abstract]
#14	Intervention, Percutaneous Coronary[Title/Abstract]
#15	Interventions, Percutaneous Coronary[Title/Abstract]
#16	Percutaneous Coronary Interventions[Title/Abstract]
#17	Percutaneous Coronary Revascularizations[Title/Abstract]
#18	Revascularization, Percutaneous Coronary[Title/Abstract]
#19	Revascularizations, Percutaneous Coronary[Title/Abstract]
#20	PCI[Title/Abstract]
#21	or/8–20
#22	Risk factor[Title/Abstract]
#23	Risk assessment[Title/Abstract]
#24	Multivariate analysis[Title/Abstract]
#25	Multivariable logistic regression[Title/Abstract]
#26	or/22–25
#27	#1 and #7 and #21 and #26

### 2.4 Study selection

**Figure 1:**
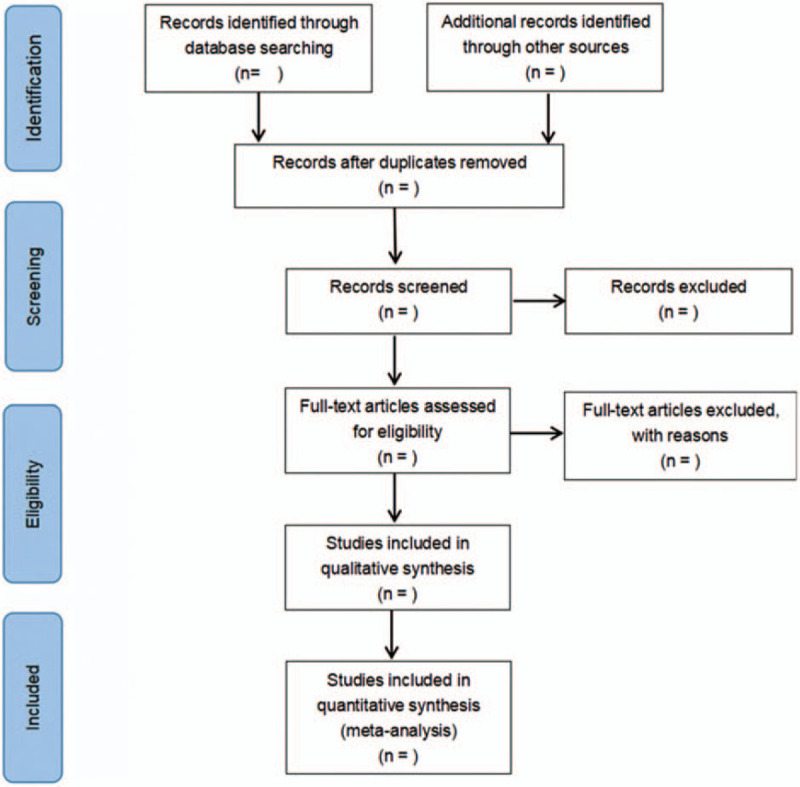
Flow diagram of literature retrieval.

First of all, the original literature was screened by 2 researchers, and a third researcher judged whether to include the literature when having conflict opinions. Secondly, the full text was re-screened on the basis of the detailed entries of the literature inclusion criteria. Finally, the selected literature was analyzed by meta-analysis. The process of the selection is exhibited in Figure 1.

### 2.5 Data extraction

The literature data were cross-checked by 2 researchers and then imported into NoteExpress for collation. The main extraction contents include first author, year of publication, country, research type, study sample size, incidence of ISR, and risk factors.

### 2.6 Assessment of the risk of bias

Two authors will independently assess the quality of selected articles using the Newcastle-Ottawa Scale (NOS).^[25]^ NOS score ≥ 5 means that the literature quality is better.

### 2.7 Data analysis

The data analysis of this study will be conducted through Review Manager Version 5.3 software. We will use OR and 95%CI to represent. If there are no findings of statistical heterogeneity, the fixed-effect model is adopted for data synthesis. If there is significant statistical heterogeneity, we will apply the random effect model. All participants will explore the possible causes from a clinical and methodological perspective and provide a descriptive or subgroup analysis.

### 2.8 Assessment of heterogeneity

The heterogeneity included in the results of the study was analyzed by performing the *χ*^2^ test (the test level was α = 0.1) and combined with *I*^2^ to quantitatively determine the size of the heterogeneity. When *P* < .1 and/or *I*^2^ > 50%, the random effect model is adopted for the combined analysis. Otherwise, the fixed-effect model is used for the combined analysis.

### 2.9 Subgroup analysis

According to the type and region of included literature, we make a subgroup analysis

### 2.10 Sensitivity analysis

To determine the stability of the outcome measures, each outcome measure was analyzed by performing sensitivity analysis.

### 2.11 Assessment of reporting biases

We will evaluate the possibility of publication bias using funnel plots and Egger's test of bias will be took as a complement.^[26]^

### 2.12 Confidence in cumulative evidence

We will evaluate the strength of evidence for all outcomes by performing the Grading of Recommendations Assessment, Development and Evaluation working group methodology.^[27]^

### 2.13 Management of missing data

We will try our best to ensure the integrity of the data. If the included data is not complete, we will take every effort to contact the corresponding author of the article, including sending emails or making a phone call. If the corresponding author cannot be contacted, we will remove the experiment with incomplete data. After data integrity is assured, intention analysis therapy and sensitivity analysis will be performed.

### 2.14 Ethical review and informed consent of patients

The content of this article does not involve moral approval or ethical review and will be presented in print or at relevant conferences.

## 3 Discussion

DM is an equal-risk condition of CHD. The prognosis of CHD patients complicated with DM was worse and the fatality rate was higher.^[28,29]^ The results of a large sample meta-analysis showed that DM was an independent risk factor of ISR.^[14]^ Abnormal glucose metabolism in DM patients often leads to insufficient insulin secretion or insulin resistance, which impairs the structure and function of vascular endothelial cells.^[30]^ Endothelial damage will stimulate the production of a large number of growth factors, and accelerate the proliferation of smooth muscle cells and inflammatory cells, thus promoting the proliferation of coronary artery intima.^[31]^ At the same time, the coagulation function of patients with DM is disordered, and platelets are easy to adhere to the damaged vascular endothelium to form thrombus, thereby narrowing the vascular lumen and the formation of ISR.^[32]^ Therefore, it is very necessary to study the risk factors of ISR in DM patients, a subgroup of high-risk groups.

At present, the research on ISR in DM patients after PCI at home and abroad is mainly single-factor and single-center research, while large sample size and multi-center research are few, and the conclusions are different. Therefore, this paper carries out meta-analysis on the risk factors of ISR in DM patients after PCI to provide clinical basis for early prevention of ISR in DM patients after PCI.

This study has the following limitations: there are differences in race, number of cases, research tools and regions of this study, and there is a certain heterogeneity after the combination of some risk factors. The purpose of this system review and meta-analysis is to clearly identify important risk factors for ISR in DM patients after PCI, thus providing prevention strategies. Most importantly, this study will assess new and controversial factors because of their potential as prevention targets.

## Author contributions

**Conceptualization:** Suiping Li and Haimei Chen.

**Data collection:** Suiping Li.

**Data curation:** Suiping Li.

**Formal analysis:** Chao Luo.

**Funding acquisition:** Haimei Chen.

**Investigation:** Chao Luo.

**Project administration:** Haimei Chen.

**Resources:** Haimei Chen.

**Software:** Chao Luo.

**Supervision:** Chao Luo, Haimei Chen.

**Validation:** Chao Luo.

**Writing – original draft:** Suiping Li and Haimei Chen.

**Writing – review & editing:** Suiping Li and Haimei Chen.
